# Neutralization of the induced VEGF-A potentiates the therapeutic effect of an anti-VEGFR2 antibody on gastric cancer in vivo

**DOI:** 10.1038/s41598-021-94584-9

**Published:** 2021-07-23

**Authors:** Tetsuo Mashima, Takeru Wakatsuki, Naomi Kawata, Myung-Kyu Jang, Akiko Nagamori, Haruka Yoshida, Kenichi Nakamura, Toshiro Migita, Hiroyuki Seimiya, Kensei Yamaguchi

**Affiliations:** 1 Division of Molecular Biotherapy, Cancer Chemotherapy Center, Japanese Foundation for Cancer Research, 3-8-31 Ariake, Koto-ku, Tokyo, 135-8550 Japan; 2 Gastroenterological Medicine, Cancer Institute Hospital, Japanese Foundation for Cancer Research, Tokyo, Japan; 3 Department of Computational Biology and Medical Sciences, Graduate School of Frontier Sciences, The University of Tokyo, Tokyo, Japan; 4 Eli Lilly Japan K.K., Kobe, Japan; 5 Tokyo Nephrology Clinic, Tokyo, Japan

**Keywords:** Preclinical research, Translational research, Cancer therapy

## Abstract

The vascular endothelial growth factor (VEGF)/VEGF receptor (VEGFR) axis is an essential regulator of angiogenesis and important therapeutic target in cancer. Ramucirumab is an anti-VEGFR2 monoclonal antibody used for the treatment of several cancers. Increased circulating VEGF-A levels after ramucirumab administration are associated with a worse prognosis, suggesting that excess VEGF-A induced by ramucirumab negatively affects treatment efficacy and that neutralizing VEGF-A may improve treatment outcomes. Here, we evaluated the effect of combination treatment with an anti-VEGFR2 antibody and anti-VEGF-A antibody on gastric tumor progression and normal tissues using a preclinical BALB/c-nu/nu mouse xenograft model. After anti-VEGFR2 antibody treatment in mice, a significant increase in plasma VEGF-A levels was observed, mirroring the clinical response. The elevated VEGF-A was host-derived. Anti-VEGF-A antibody co-administration enhanced the anti-tumor effect of the anti-VEGFR2-antibody without exacerbating the toxicity. Mechanistically, the combination treatment induced intra-tumor molecular changes closely related to angiogenesis inhibition and abolished the gene expression changes specifically induced by anti-VEGFR2 antibody treatment alone. We particularly identified the dual treatment-selective downregulation of *ZEB1* expression, which was critical for gastric cancer cell proliferation. These data indicate that the dual blockade of VEGF-A and VEGFR2 is a rational strategy to ensure the anti-tumor effect of angiogenesis-targeting therapy.

## Introduction

Gastric cancer is the seventh most commonly diagnosed cancer and the third leading cause of cancer-related deaths worldwide^1^. In Japan, gastric cancer is currently the second most frequently diagnosed cancer and the third leading cause of cancer-related deaths (Japanese nationwide cancer registry; https://ganjoho.jp/reg_stat/index.html). In advanced gastric cancer, systemic chemotherapy with fluoropyrimidine plus a platinum agent is the standard of care worldwide^2,3^. Moreover, several biologics, such as anti-angiogenic agents and immune checkpoint inhibitors, have been widely used for gastric cancer^4,5^. However, these treatments have shown limited patient benefits, and the prognosis of advanced gastric cancer remains poor with a median overall survival of less than 1 year globally^6,7^. Therefore, new therapeutic options are required to achieve better treatment outcomes.

Vascular endothelial growth factor-A (VEGF-A) derived from tumors and host mesenchymal tissues plays an essential role in tumor progression by promoting angiogenesis mainly through its receptor VEGF receptor 2 (VEGFR2) expressed in vascular endothelial cells^8^. Therefore, the pharmacological blockade of this pathway with antibodies, recombinant proteins, and compounds has been approved for the treatment of solid tumors^8,9^. Ramucirumab is a fully humanized anti-VEGFR2 monoclonal antibody^10^. The randomized phase 3 RAINBOW study comparing paclitaxel plus ramucirumab and paclitaxel plus placebo showed a significantly prolonged overall survival from 7.4 months in the placebo group to 9.6 months in the ramucirumab group. Based on these data, ramucirumab was approved and is currently widely used as a second-line treatment in gastric cancer^4^.

Several previous studies have reported that pharmacodynamic (PD) changes are associated with the treatment efficacy of anti-angiogenic agents^11,12^. Specifically, a marked increase in plasma VEGF-A levels was observed after the administration of ramucirumab or DC101, a mouse surrogate anti-VEGFR2 antibody^13,14^. In addition, highly increased VEGF-A concentrations after ramucirumab administration^15^ as well as elevated basal plasma levels of VEGF-A^16^ was shown to be associated with worse prognosis in gastric cancer. In our previous clinical study^15^, the elevation of VEGF-A after ramucirumab treatment was predominantly associated with worse outcome among the VEGF members that we analyzed. Based on these observations, we hypothesized that excess VEGF-A induced by ramucirumab negatively affects treatment efficacy and that neutralizing VEGF-A can improve treatment outcomes. Here, we examined the therapeutic significance of anti-VEGF-A antibody co-administration in VEGFR2-targeted therapy using a preclinical mouse xenograft model.

## Materials and methods

### Cell lines and cell culture

The human gastric cancer cell lines used in this study were obtained from JFCR39, the Japanese cell line panel, and cultured, as described previously^17^. Cells were maintained in RPMI1640 medium supplemented with 10% heat-inactivated fetal bovine serum and 100 μg/ml of kanamycin in a humidified atmosphere of 5% CO_2_ and 95% air at 37 °C.

### Antibodies

The anti-mouse VEGFR2 antibody DC101^13^ was purchased from Bio X Cell (Lebanon, NH). The anti-mouse VEGF-A antibody 2G11-2A05, which recognizes both mouse and human VEGF-A and possesses neutralizing potential^18,19^, was obtained from BioLegend (San Diego, CA).

### Enzyme-linked immunosorbent assay (ELISA)

Mouse and human VEGF-A, VEGF-C, VEGF-D, and placental growth factor (PlGF) levels in murine plasma were measured using Quantikine ELISA kits (R&D Systems, Minneapolis, MN) according to the manufacturer’s instructions^20^. Samples were measured in triplicate. To measure free VEGF-A (unbound to anti-VEGF-A antibody) concentrations, plasma samples were immune depleted using Dynabeads Protein G (Thermo Fisher Scientific, Waltham, MA) following protocols described previously^21,22^.

### Therapeutic study with mouse xenograft model and pathological analysis

All preclinical studies in the mouse xenograft model were performed in the Japanese Foundation for Cancer Research (JFCR) animal experiment room. The protocols were approved by the JFCR Animal Care and Use Committee and in compliance with ARRIVE guidelines as described previously^23^. Experimental conditions and procedures in detail are described in the Supplementary Materials and Methods. We defined a body-weight reduction of 20% as a parameter for humane endpoint decisions and performed the experiments within the criteria. At the end of the experiments, mice were euthanized by cervical dislocation. Five-week-old female nude mice with a BALB/c genetic background (Charles River Laboratories, Yokohama, Japan) were subcutaneously injected with 3 × 10^6^ MKN45 cells. When tumors reached 100–200 mm^3^, the mice were divided into each treatment group and a vehicle (PBS) group. The anti-mouse VEGFR2 antibody (DC101) (10 or 20 mg/kg), anti-mouse VEGF-A antibody (2G11-2A05) (5 mg/kg), and the two antibodies together were administered intraperitoneally twice a week for 2 weeks to examine their therapeutic effect. We determined the dose of the antibodies and treatment interval based on the previous studies with the antibodies^13,18,19^. The length (L) and width (W) of each tumor was measured using a digital caliper, and the tumor volume was calculated as (L × W^2^)/2. To prepare mouse plasma, blood samples collected with EDTA at a final concentration of 1 mg/m l were centrifuged at 2000 g for 20 min, and the supernatants were collected.

Xenograft tumors and tissue samples were obtained on day 14 after the start of antibody treatment and fixed in Mildform 10 N (Wako Pure Chemical Industries, Ltd., Osaka, Japan). All tissues were embedded in paraffin, and histological sections were stained with hematoxylin and eosin (H&E) or Periodic acid–Schiff (PAS) as described previously^24^.

### cDNA microarray and gene signature-based pathway analysis

Xenograft tumor tissues were collected on day 14 after the start of antibody treatment and preserved in Allprotect Tissue Reagent (Qiagen, Hilden, Germany). Total RNA was extracted from tumor tissues with an RNeasy Mini kit (Qiagen) and TissueLyser (Qiagen) and quantified using an Agilent 2100 Bioanalyzer (Agilent Technologies, Santa Clara, CA). cDNA microarray analysis was performed with the GeneChip™ Human Genome U133 Plus 2.0 Array (Thermo Fisher Scientific) according to the manufacturer’s protocol. Data were normalized by MAS5 method and hierarchical clustering was performed using GeneSpring GX software (Agilent Technologies). Metascape analysis was conducted on the website (https://metascape.org/gp/index.html). Gene Ontology (GO) analysis and KEGG pathway analysis was performed on the DAVID website (https://david.ncifcrf.gov/). The gene expression data were deposited in Gene Expression Omnibus (GEO) and are accessible through the accession number GSE 160613. The data will be released on January 1, 2022.

### Reverse transcription-quantitative PCR (RT-qPCR)

Total RNA was extracted with an RNeasy Mini kit (Qiagen). cDNA was synthesized with SuperScript III First-Strand Synthesis SuperMix for RT-qPCR (Life Technologies, Carlsbad, CA). RT-qPCR was performed using a LightCycler 96 (Roche, Basel, Switzerland). Primer sequences for RT-qPCR were as follows: *ZEB1* forward primer: 5′-TTTCCTGAGGCACCTGAAGAG-3′, *ZEB1* reverse primer: 5′-TGGACAGGTGAGTAATTGTG-3′, *MECP2* forward primer: 5′-GAAAAGATGGGCAGCACGC-3′, *MECP2* reverse primer: 5′-GGTGCAAACGCGTCACTTAG-3′, *GAPDH* forward primer: 5′-GAAGGTGAAGGTCGGAGTC-3′, *GAPDH* reverse primer: 5′-GAAGATGGTGATGGGATTTC-3′.

### siRNA treatment and cell proliferation assay

Silencer select siRNAs and negative control siRNA were purchased from Thermo Fisher Scientific. We introduced the siRNAs into cells with Lipofectamine RNAiMAX Transfection Reagent (Thermo Fisher Scientific). The siRNAs used in this study are as follows: siRNA against *ZEB1 #1* (5′-CAGUCUGGGUGUAAUCGUAtt-3′), siRNA against *ZEB1 #2* (5′-GAACUUGUCUUGCGCAAAAtt-3′). After a 3-day incubation, *ZEB1* knockdown was examined by RT-qPCR. At the same time, cells were seeded in 96-well microplates and we continued cell culture for indicated time periods. For the cell viability measurement, thiazolyl blue tetrazolium bromide (MTT) was added to the medium at a final concentration of 0.5 mg/mL. After incubation for 4 h, the medium containing MTT was removed and dimethyl sulfoxide was added. Optical density at 570 nm and 630 nm (reference) was measured.

### Statistical analysis

Statistical significance was evaluated by ANOVA, followed by the Tukey–Kramer post-hoc test.

### Ethics approval and consent to participate

All animal procedures were performed in accordance with the national laws and policies (Guidelines for Proper Conduct of Animal Experiments, Science Council of Japan, 2006). The procedures were conducted in the animal experiment room at JFCR using protocols that were approved by the JFCR Animal Care and Use Committee.

## Results

### Preclinical mouse xenograft model mimics VEGF elevation in blood after anti-VEGFR2-targeted therapy

**Figure 1:**
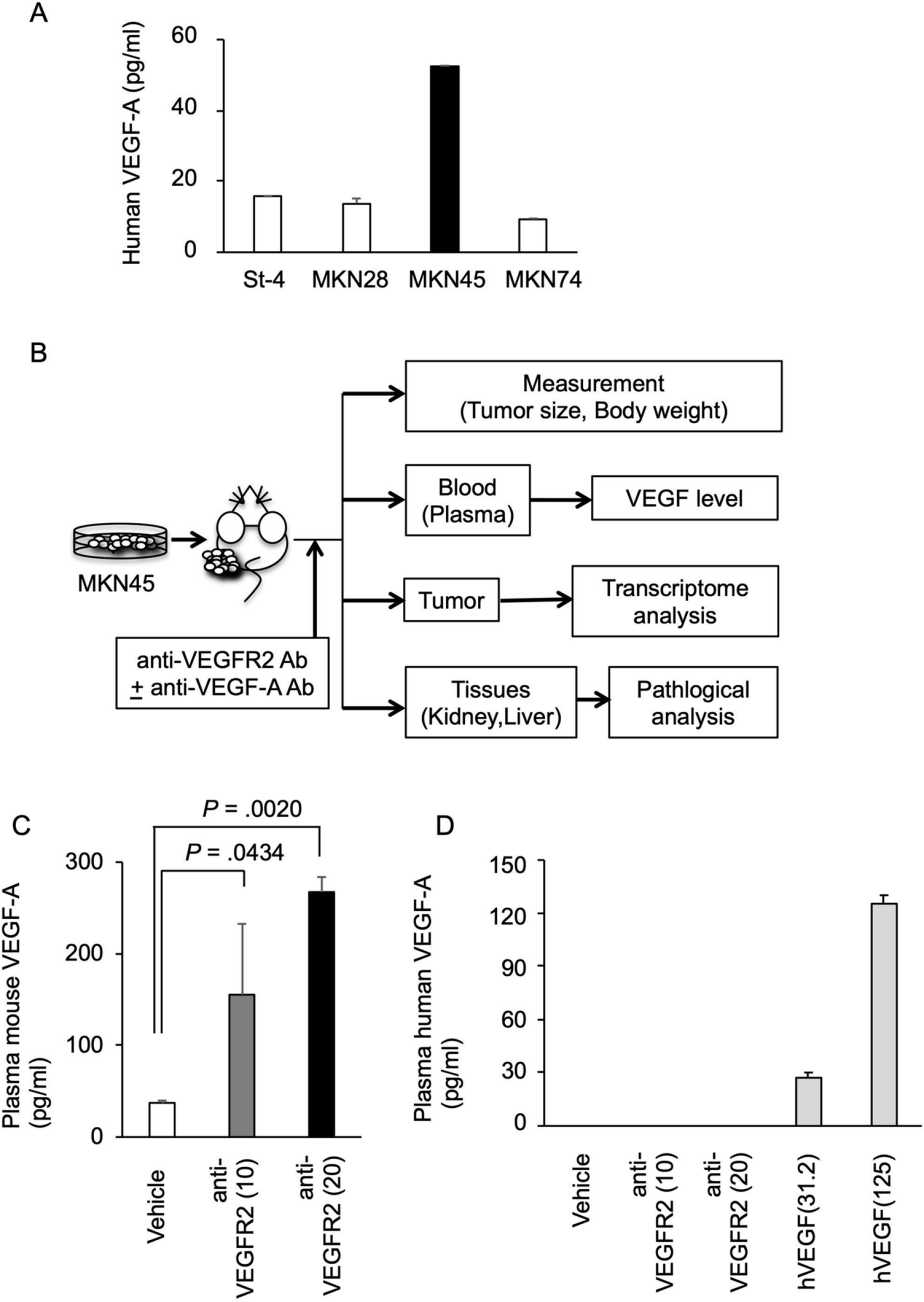
Elevated host-derived VEGF-A in the blood after VEGFR2-targeted therapy in a gastric cancer xenograft model. (**A**) Human VEGF-A levels secreted by human gastric cancer cell lines. Human gastric cancer cell lines were cultured for 72 h, and conditioned media were collected. Human VEGF-A level in the conditioned medium was measured as described in Materials and Methods. The VEGF-A concentrations normalized by cell number (per 1 × 10^4^ cells) are shown. (**B**) Schematic diagram of the evaluation of plasma VEGF levels and therapeutic effect of VEGFR2 and VEGF-A dual targeting in a gastric cancer xenograft model. (**C**,**D**) Alterations in murine (**C**) and human (**D**) VEGF-A levels in mouse plasma following anti-mouse VEGFR2 antibody administration were shown. BALB/c nude mice were subcutaneously injected with 3 × 10^6^ MKN45 cells. When the tumor reached 100–200 mm^3^, mice were intraperitoneally treated with vehicle (PBS) or an anti-mouse VEGFR2 antibody (DC101) (10 or 20 mg/kg) twice a week for 2 weeks (N = 3). At 14 days after the start of treatment, mouse plasma was collected, and human and murine VEGF-A concentrations were measured as described in Materials and Methods. For the estimation of human VEGF-A levels, purified human VEGF-A protein solutions (hVEGF, 31.2 and 125 pg/ml) were also tested as controls. Statistical significance was evaluated by ANOVA, followed by the Tukey–Kramer post-hoc test. The figures were generated by Microsoft Powerpoint (16.16.27) (https://www.microsoft.com/ja-jp/microsoft-365/powerpoint).

To examine the effects of VEGF-A inhibition on the therapeutic potential of VEGFR2-targeted therapy, we used a mouse xenograft model inoculated with human gastric cancer cells according to previous preclinical evaluations of VEGFR2-targeted agents^13^, which were based on the evidence that there is minimal species-specificity in the effect of VEGF on VEGFR^25^. To select a suitable gastric cancer cell line for analysis, we first measured VEGF-A production in human gastric cancer cell lines. As shown in Fig. 1A, all tested gastric cancer cells secreted VEGF-A, and the highest levels were detected in MKN45 cells. Based on these data, we subcutaneously transplanted MKN45 cells into the xenograft model for further analysis (Fig. 1B). In this model, we used the anti-mouse VEGFR2 antibody DC101 as a surrogate antibody for ramucirumab^13^, which targets VEGFR2 mainly expressed in vascular endothelial cells. Using this xenograft model, we examined whether the levels of VEGF-A and other soluble factors in the blood were induced after anti-VEGFR2 antibody administration, as observed in clinical patients^14^. We also determined whether the induced factors were derived from host tissues or human tumors using species-selective ELISAs, which clearly distinguish between mouse- and human-derived factors (Suppl Fig. 1). As shown in Fig. 1C, murine VEGF-A levels were strongly induced in mouse plasma after DC101 administration. This elevation in murine VEGF-A was also observed in mice not harboring MKN45 tumors (Suppl Fig. 2A). In contrast, human VEGF-A was not detected in the plasma after DC101 treatment (Fig. 1D). Similarly, mouse PlGF was induced in the plasma after DC101 administration (Suppl Fig. 2B). In addition, mouse VEGF-C and VEGF-D were slightly elevated after DC101 treatment (Suppl Fig. 2C,D), whereas human PlGF, VEGF-C, and VEGF-D were not detected in the plasma (data not shown). These data indicate that our xenograft model mimics the PD changes observed in clinical patients. Moreover, we showed that the elevated levels of VEGF-A and other factors in the blood after anti-VEGFR2 antibody administration were mainly host tissue-derived.

### Co-administration of an anti-VEGF-A antibody potentiates the anti-tumor effect of anti-VEGFR2 antibody treatment in a gastric cancer xenograft model

**Figure 2:**
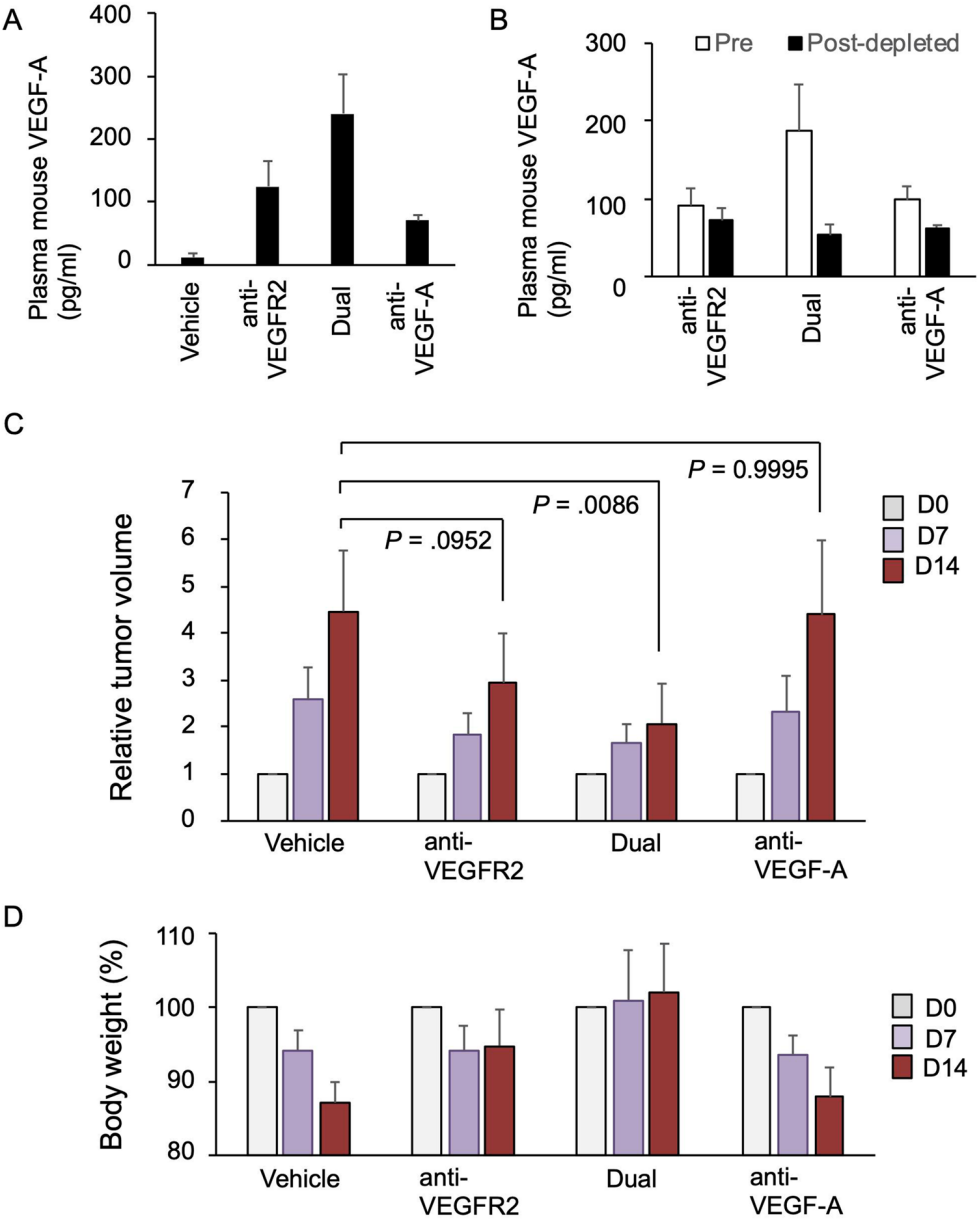
Effect of the co-administration of a VEGF-A neutralizing antibody and VEGFR2-targeted antibody in a gastric cancer xenograft model. (**A**) Plasma VEGF-A levels after anti-VEGFR2 antibody and/or anti-VEGF-A antibody treatment. BALB/c nude mice were injected with MKN45 cells. When the tumor reached 100–200 mm^3^, the mice were intraperitoneally treated with vehicle (PBS), an anti-mouse VEGFR2 antibody (10 mg/kg), an anti-mouse VEGF-A antibody (5 mg/kg), and the two antibodies together (Dual) twice a week for 2 weeks (N = 3). At 14 days after the start of treatment, mouse plasma was collected, and the murine VEGF-A concentration was measured. (**B**) Comparison of total and free VEGF-A (unbound to antibody) levels in the plasma of mice treated with the anti-VEGFR2 antibody alone or together with the anti-VEGF-A antibody (Dual). BALB/c nude mice were injected with MKN45 cells, treated with the anti-mouse VEGFR2 antibody (10 mg/kg) alone or together with the anti-mouse VEGF-A antibody, and murine plasma was collected as in (**A**). The plasma was incubated in the presence or absence of protein-G beads to deplete the antibody-bound fraction, and mouse VEGF-A levels were measured as described in Materials and Methods. (**C**,**D**) BALB/c nude mice were injected with MKN45 cells. When the tumor reached 100–200 mm^3^, the mice were treated with the vehicle (N = 7), anti-VEGFR2 antibody (10 mg/kg) alone (N = 8), anti-VEGFR2 antibody (10 mg/kg) and anti-VEGF-A antibody (5 mg/kg) together (Dual, N = 6), and anti-VEGF-A antibody (5 mg/kg) alone (N = 5). The length (L) and width (W) of each tumor was measured at days 0, 7, and 14 using a digital caliper, and the tumor volume was calculated as (L × W^2^)/2. Relative tumor volume was shown in (**C**). Statistical significance was estimated by ANOVA, followed by the Tukey–Kramer post-hoc test. Body weight changes in mice in the treatment groups were indicated in (**D**). The figures were generated by Microsoft Powerpoint (16.16.27) (https://www.microsoft.com/ja-jp/microsoft-365/powerpoint).

To determine the significance of VEGF-A in the therapeutic effect of the anti-VEGFR2 antibody, we tested the co-administration of an anti-VEGF-A antibody and anti-VEGFR2 antibody. For this purpose, we used the anti-mouse VEGF-A antibody 2G11-2A05, which can neutralize mouse VEGF-A activity^18^. Before initiating the combination treatment, we performed a pilot time course experiment, which revealed that the elevation in plasma VEGF-A was observed at early time points after anti-VEGFR2 antibody administration (starting at 24 h) (Suppl Fig. 2A). Based on these data and previous preclinical in vivo studies using this antibody^19^, we co-administrated the anti-mouse VEGF-A antibody (2G11-2A05) and anti-mouse VEGFR2 antibody (DC101) to our xenograft model. When we treated mice with a combination of the anti-VEGF-A antibody and the anti-VEGFR2 antibody (indicated as ‘Dual’ in Figures), we observed a greater increase in mouse VEGF-A in the plasma, but the elevated VEGF-A was mainly the antibody-captured fraction (Fig. 2A,B). These data suggest that most of the circulating VEGF-A could be captured by the anti-VEGF-A antibody, as shown in previous studies^21,22^.

**Figure 3:**
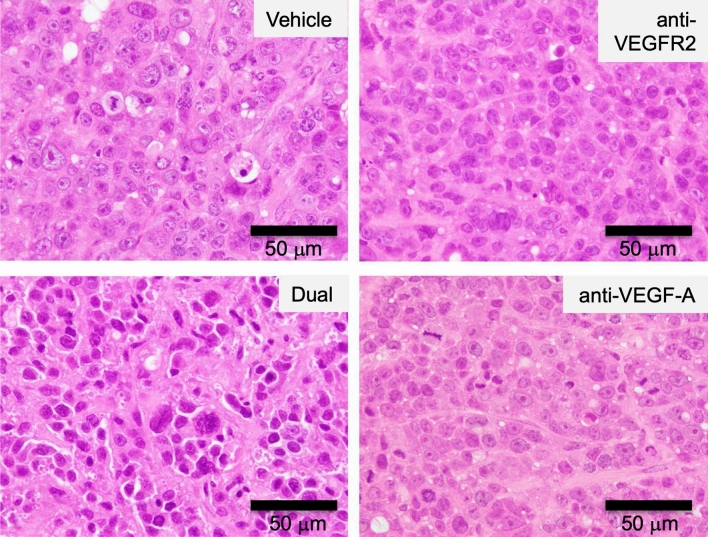
Histological analysis of xenograft tumors after dual treatment with anti-VEGFR2 and anti-VEGF-A antibodies. After each treatment, xenograft tumor tissues were fixed, embedded in paraffin, and stained with hematoxylin and eosin (H&E) as described in Materials and Methods. Typical staining results in each treatment group were shown. Original magnification, ×40. The figures were generated by Microsoft Powerpoint (16.16.27) (https://www.microsoft.com/ja-jp/microsoft-365/powerpoint).

We further tested the cooperative effect of anti-VEGF-A antibody and the anti-VEGFR2 antibody in the xenograft model. Before treatment, each group of mice showed similar body weight (17–20 g). The co-treatment with these antibodies showed an enhanced anti-tumor effect on xenograft tumors (Fig. 2C). Histological analysis of xenograft tumors revealed significant effect of the treatments with these antibodies (Fig. 3). Cancer cells with vehicle treatment showed cellular heterogeneity which is characterized by marked variation of nuclear size with prominent nucleoli and frequent mitoses. Cancer cells proliferate in a solid pattern. In contrast to the vehicle treatment, cancer cells with antibody treatments represent less cellular heterogeneity. In particular, cancer cells with the dual treatment have atypical hyperchromatic nuclei with indistinct nucleoli. Also, there is an increased fibrous stroma in the xenograft tumor with the dual treatment. Additionally, we also observed a tendency of decrease in the Ki67-positive cancer cells in the dual treatment (50 ± 15.8%) compared with anti-VEGFR2 single treatment (69.8 ± 9.8%) (Suppl Table 1 and Suppl Fig. 3). During treatment, no marked decrease in the body weight of mice was observed (Fig. 2D).

**Figure 4:**
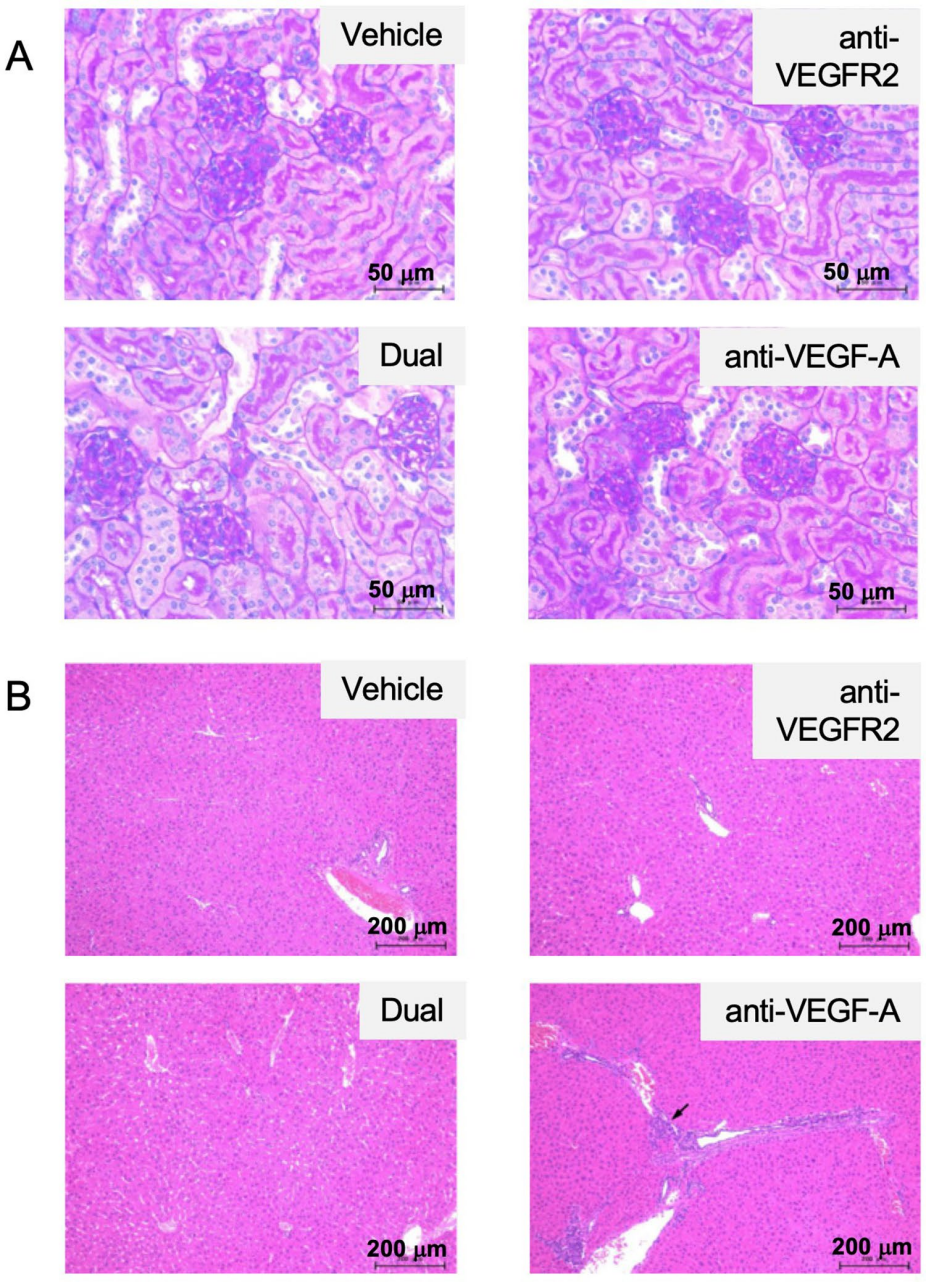
Histological analysis of organs after dual treatment with anti-VEGFR2 and anti-VEGF-A antibodies in the xenograft model. (**A**) Effect of treatment with anti-VEGFR2 and anti-VEGF-A antibodies on mouse kidneys. After each treatment, kidney tissues were fixed, embedded in paraffin, and stained with Periodic acid–Schiff (PAS) as described in Materials and Methods. Typical staining results in each treatment group were shown. Original magnification, ×40. (**B**) Effect of treatment with anti-VEGFR2 and anti-VEGF-A antibodies on mouse livers. Liver tissues were fixed, embedded in paraffin, and stained with hematoxylin and eosin (H&E) as described in Materials and Methods. Typical staining results in each treatment group were shown. The arrow indicates slight bile duct hyperplasia. Original magnification, ×10. The figures were generated by Microsoft Powerpoint (16.16.27) (https://www.microsoft.com/ja-jp/microsoft-365/powerpoint).

To assess the adverse effects on mice in more detail, pathological analyses of the kidney and liver were performed. We observed a marginal level of infarction and atrophic tubules in the kidney of some treatment- and vehicle-treated mice and a marginal level of bile duct hyperplasia in the liver of an anti-VEGF-A antibody-treated mouse (Suppl Table 2). However, no apparently enhanced pathological changes in kidney (particularly in the glomerulus and tubules) or liver tissues were observed after the combination treatment (Fig. 4A,B). These results indicate that anti-VEGF-A antibody co-treatment may be an effective strategy to ensure the anti-tumor effect of VEGFR2-targeted therapy without exacerbating the toxicity.

### Molecular pathways affected by the combination treatment in xenograft tumors

**Figure 5:**
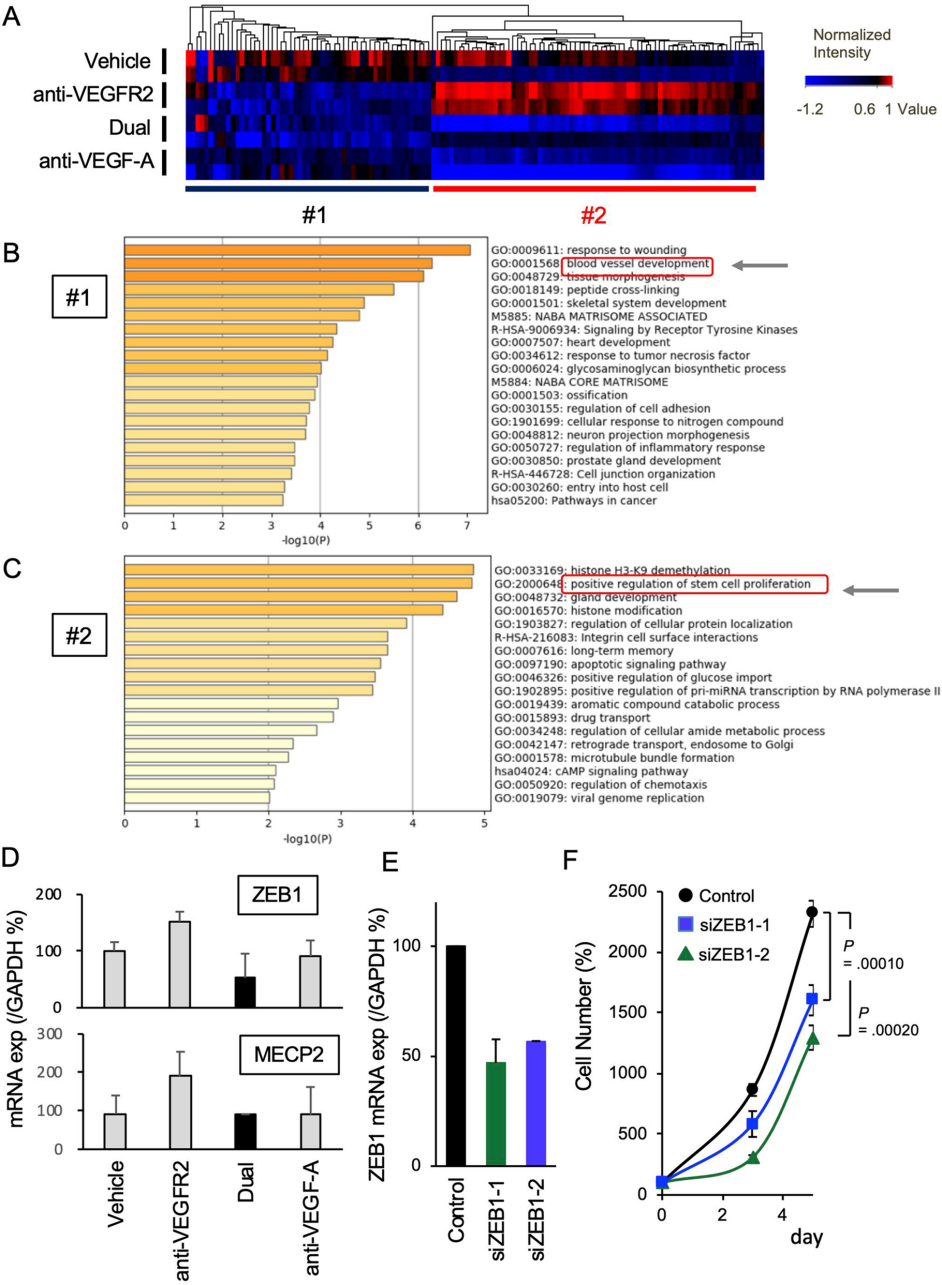
Transcriptome analysis of gastric cancer xenografts treated with anti-VEGFR2 and anti-VEGF-A antibodies. (**A**) BALB/c nude mice were injected with MKN45 cells and treated with each agent as in Fig. 2(C) (N = 2). Xenograft tumor tissues were collected on day 14 after the start of antibody treatment, and cDNA microarray analysis was performed as described in Materials and Methods. Hierarchical clustering of genes with altered expression in the VEGFR2 and VEGF-A targeted antibody-treated xenograft tumors was performed. Genes showing > 1.5-fold changes in expression by the antibody treatment were extracted, and hierarchical clustering was performed as described in Materials and Methods. (**B**) Metascape analysis (https://metascape.org/gp/index.html) of the genes downregulated by anti-VEGFR2 antibody treatment (classified as #1 in (**A**)) was performed. For the analysis, we extracted and analyzed the gene sets that were > 40% downregulated by anti-VEGFR2 antibody treatment compared with the vehicle control. (**C**) Metascape analysis of the genes upregulated by anti-VEGFR2 antibody treatment (classified as #2 in (**A**)) was performed. For the analysis, we extracted and analyzed the gene sets that were > 60% upregulated by anti-VEGFR2 antibody treatment but not by the anti-VEGF-A antibody compared with the vehicle control. (**D**) mRNA expression of *ZEB1* and *MECP2* in xenograft tumors after each treatment. BALB/c nude mice were injected with MKN45 cells and treated with each agent as in (**A**) (N = 3). Xenograft tumor tissues were collected on day 14 after the start of antibody treatment, and mRNA expression was examined by reverse transcription -quantitative PCR (RT-qPCR). (**E)** *ZEB1* knockdown by siRNAs in MKN45 cells. Cells were transfected with the *ZEB1* siRNAs or control siRNA. After a 3-day incubation, the *ZEB1* expression was exaimined by RT-qPCR. (**F**) Anti-proliferative effect of *ZEB1* knockdown in MKN45 cells. After the treatment of cells with siRNAs as in (**E**), cells were cultured for indicated time periods. Cell viability was determined as described in Materials and Methods. The figures were generated by Microsoft Powerpoint (16.16.27) (https://www.microsoft.com/ja-jp/microsoft-365/powerpoint).

To gain further insight into the molecular pathways by which the antibody combination affects tumor tissues, transcriptome analysis was performed in tumor xenografts on day 14 following each treatment. Hierarchical clustering analysis revealed that genes affected by the treatment were classified into two classes: those that were commonly downregulated by the anti-VEGFR2 antibody and anti-VEGF-A antibody (#1 in Fig. 5A) and those that were inversely regulated by these treatments (#2 in Fig. 5A). Metascape analysis of the genes downregulated by anti-VEGFR2 antibody treatment (corresponding to #1 in Fig. 5A) revealed that these genes were closely related to angiogenesis (Fig. 5B). These genes were strongly suppressed by the co-administration of the two antibodies, suggesting that VEGF-A/VEGFR2-dependent angiogenesis could be inhibited by the combination therapy. In contrast, Metascape analysis on the genes specifically upregulated by anti-VEGFR2 antibody treatment (corresponding to #2 in Fig. 5A) showed that these genes were related to histone modification and the regulation of stem cell functions associated with tumor progression (Fig. 5C). The Gene Ontology (GO) and KEGG pathway search on the DAVID analysis further suggested additional molecular pathways related to the #2 gene, including mRNA regulation or stemness/cancer-related signaling pathways (Suppl Tables 3 and 4). Among the genes differentially regulated between anti-VEGFR2 alone and the dual treatment, we focused on the genes involved in stem cell proliferation, since stemness is associated with cancer progression^26^. By q-RT-PCR analysis, we particularly confirmed the differential expression of *ZEB1* and *MECP2*, the genes involved in cancer stemness and proliferation^27,28^. We found that the dual treatment preferentially suppressed *ZEB1* expression (Fig. 5D). Moreover, the knockdown of *ZEB1* significantly inhibited proliferation of MKN45 cells (Fig. 5E,F). These results suggest that *ZEB1* downregulation could be a critical step for the dual treatment-mediated tumor suppression.

**Figure 6:**
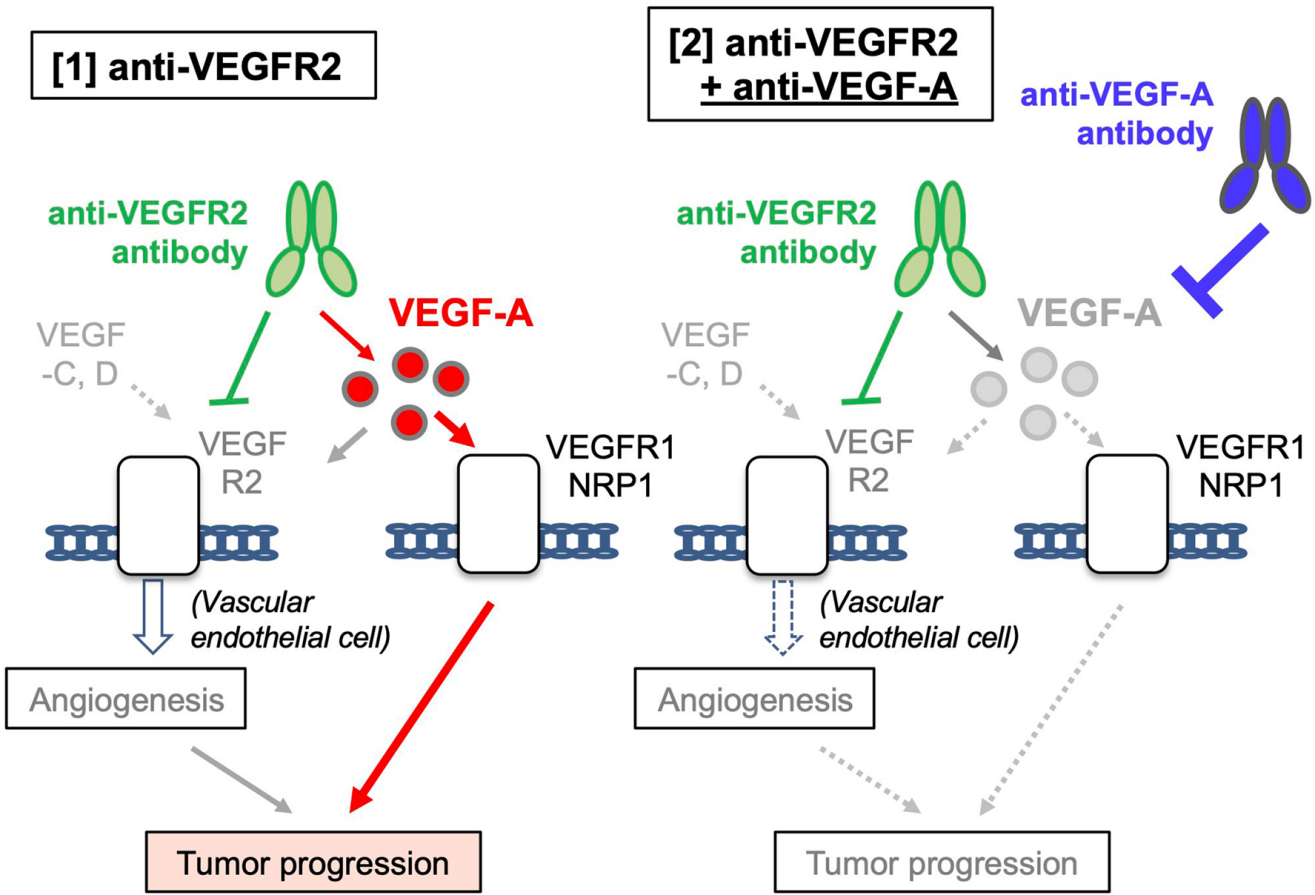
Possible molecular pathways affected by VEGFR2 and VEGF-A targeted therapy. The figures were generated by Microsoft Powerpoint (16.16.27) (https://www.microsoft.com/ja-jp/microsoft-365/powerpoint).

Collectively, these data indicate that the ligand/receptor dual-targeting of this pathway ensures target-specific anti-angiogenic effects to suppress tumor progression while compensating for other alterations in cell signaling (Summarized in Fig. 6).

## Discussion

VEGF/VEGFR pathways are regulated by multiple ligands and receptors^8^. This redundancy indicates that angiogenesis is a physiologically vital phenomenon that will likely be conserved against any interference of this pathway. In other words, this complex regulation by multiple factors may be implicated in the acquired resistance of tumors to angiogenic inhibitors. To overcome this redundant regulation system, combination treatments, such as ligand-receptor dual blockade, provides a promising strategy. In this study, we demonstrated that the dual blockade of the VEGF-A/VEGFR2 pathway using anti-VEGFR2 and anti-VEGF-A antibodies significantly suppressed gastric tumor growth in a preclinical mouse model. According to gene expression analysis, this dual blockade therapy strongly suppressed the expression of genes related to angiogenesis and other tumor-promoting pathways compared with the anti-VEGFR2 antibody alone. The preclinical mouse xenograft model mirroring the clinical response was valuable to evaluate the novel combination treatment. To the best of our knowledge, our study is the first to describe this potential novel treatment strategy involving the dual blockade of the VEGF-A/VEGFR2 pathway using anti-VEGFR2 and anti-VEGF-A antibodies.

Recent studies have reported increased levels of VEGF-A, VEGF-C, and VEGF-D during bevacizumab treatment^29,30^. Because VEGF-C and -D function as alternative ligands to stimulate VEGFR2 when VEGF-A is inhibited, an increase in these ligands during treatment may contribute to the resistance of tumors to bevacizumab. Regarding VEGFR2 inhibition, several clinical and preclinical studies showed that the plasma VEGF-A level increased after the administration of ramucirumab or DC101^13–15^. In addition, an inverse correlation between the increased VEGF-A level and patient survival in gastric cancer was reported in patients who received ramucirumab treatment^15^. These data suggest that the elevated VEGF-A after anti-VEGFR2-targeted therapy counteracts the treatment effect and that targeting VEGF-A with a neutralizing antibody may achieve better treatment outcomes.

The exact mechanisms by which increased VEGF-A levels after anti-VEGFR2 antibody administration affect tumor progression or malignancy remain unclear. When VEGFR2 is completely inhibited, excess VEGF-A can bind to other receptors, such as VEGFR1 or neuropilin-1 (NRP-1). Previous studies have shown that VEGF-A acts on tumor-expressed VEGFR1 to promote tumorigenesis. In fact, VEGFR1 is expressed in 76% of gastric cancer tumors^31^, and VEGF-A can stimulate VEGFR1 on tumor cells leading to increased cell proliferation^32^. Additionally, VEGF-A can act on VEGFR1 or NRP-1 expressed in the tumor microenvironment of host cells to promote metastasis and immune suppression. VEGFR1 pathway activation in macrophages and monocytes is involved in their infiltration into tumors and is associated with increased liver and lung metastasis^33–36^. VEGF-A also stimulates VEGFR1 on myeloid-derived suppressor cells or NRP-1 on regulatory T cells, leading to immune tolerance^37–39^. These data suggest that excess VEGF-A cannot only promote tumor growth but also facilitate metastasis and immune-suppression by stimulating VEGFR1 and NRP-1 pathways, resulting in tumor progression. Therefore, the combination of VEGF-A neutralization and anti-VEGFR2 antibody treatment would disrupt these pathways to enhance anti-tumor activity. On the other hand, it is also likely that VEGFR2 is incompletely neutralized by the anti-VEGFR2 therapy which could further explain the added benefit of anti-VEGF.

In this study, we used a nude mouse xenograft model, which possesses B cells, natural killer (NK) cells, macrophages, and dendritic cells but lacks T cells. Therefore, this system could not be used to evaluate the potential effect of VEGF-A neutralization on T cell-related immunosuppression. Further analysis with immune competent mice and murine cancer cell lines such as MFC cells^40^ is needed to determine the effect of the combination therapy in more detail. With such in vivo models, efficacy of the dual treatment on tumor metastasis should also be estimated further.

Our gene expression analysis revealed that VEGFR2- and VEGF-A-targeted antibody treatments exhibited distinct effects on tumor tissues. They both suppressed angiogenesis-related genes, whereas other subsets of genes were oppositely regulated by these antibodies (Fig. 5A). We speculate that the different molecular changes may be related to the various effect of these agents on VEGF-A-dependent and VEGFR2-independent (possibly VEGF-A/VEGFR1-mediated) pathways. The increased VEGF-A induced by the anti-VEGFR2 antibody would activate these pathways, whereas anti-VEGF-A antibody administration should neutralize VEGF-A activity and suppress these pathways (Fig. 6). Of note, these genes were related to stem cell proliferation and histone modification (Fig. 5C), which are closely associated with tumor progression^26,41^. Further detailed analysis is required to uncover the molecular link between these pathways and the therapeutic effect of VEGFR2/VEGF-A antibodies.

Here, we showed a superior effect of the dual blockade of the VEGF/VEGFR2 pathway using anti-VEGFR2 and anti-VEGF-A antibodies compared with the use of each antibody alone. A previous clinical trial investigating a combination of the anti-VEGF-A antibody bevacizumab and multi-kinase inhibitor sorafenib targeting VEGFRs showed promising efficacy with a response rate of 43% in patients with ovarian cancer^42^. However, several data from clinical trials evaluating bevacizumab and multi-kinase inhibitors targeting VEGFRs have shown a trend of higher toxicity profiles^43–45^. Liu et al. reviewed 144 clinical trials and described that compromised dosing was often needed for the combination of compounds targeting overlapped pathways but not for two antibodies, especially those targeting angiogenesis^46^. So far, the kinase inhibitors targeting VEGFRs are not specific to VEGFR2, and indicated higher toxicity in combination with anti-VEGF-A antibody. In this study, we selected an anti-VEGF-A antibody and anti-VEGFR2 antibody, and the use of this combination in mice did not result in any body weight loss or severe pathologic findings in the liver and kidney.

Body weight loss during tumor progression is a typical phenotype of cachexia and gastric cancer patients have the highest incidence of cachexia^47,48^. In particular, the MKN45 cell-derived xenograft that we used in this study is a well-known model of tumor-induced cachexia^48^. Therefore, we speculate that the combined treatment did not lose weight when compared with the single treatments because the combined treatment suppressed tumor growth most effectively and prevented the MKN45 tumor-derived cachexia. Additionally, VEGF-A itself causes cachexia^49^. Therefore, the VEGF-A blockade by the dual treatment could further ameliorated cachexia to prevent body weight loss. Thus, this combination of antibodies may be preferable in dual blockade VEGF/VEGFR2 treatment, while further preclinical and clinical evaluation of its efficacy and safety including such side-effects as hypertension, hemorrhage, perforation, anemia is required.

The effective anti-tumor potential of ligand/receptor dual inhibition was also suggested in PD-1/PD-L1-targeted agents^50^. Co-administration of dual HER2-targeted agents with different binding sites was also shown to be effective in a clinical setting^51^. Together with our observations, these data suggest that the simultaneous inhibition of multiple factors in a signaling pathway is conceptually a reasonable approach to avoid the development of drug resistance and achieve effective therapeutic outcomes.

In summary, our data suggest that the dual blockade of VEGF-A and VEGFR2 is a unique and promising treatment strategy to enhance the anti-tumor effect of angiogenesis-targeted therapy. This concept should be evaluated in clinical trials and further molecular correlation analysis not only for angiogenesis but also for immunity.

## Supplementary Information

Supplementary Information.

## Data Availability

The gene expression data have been deposited in Gene Expression Omnibus (GEO) and are accessible through the accession number GSE160613. The data will be released on January 1, 2022.

## References

[1] Machlowska J, Baj J, Sitarz M, Maciejewski R, Sitarz R (2020). Gastric cancer: Epidemiology, risk factors, classification, genomic characteristics and treatment strategies. Int. J. Mol. Sci..

[2] Koizumi W, Narahara H, Hara T, Takagane A, Akiya T, Takagi M (2008). S-1 plus cisplatin versus S-1 alone for first-line treatment of advanced gastric cancer (SPIRITS trial): A phase III trial. Lancet Oncol..

[3] Yamada Y (2015). Phase III study comparing oxaliplatin plus S-1 with cisplatin plus S-1 in chemotherapy-naïve patients with advanced gastric cancer. Ann. Oncol..

[4] Wilke H (2014). Ramucirumab plus paclitaxel versus placebo plus paclitaxel in patients with previously treated advanced gastric or gastro-oesophageal junction adenocarcinoma (RAINBOW): A double-blind, randomised phase 3 trial. Lancet Oncol..

[5] Kang YK (2017). Nivolumab in patients with advanced gastric or gastro-oesophageal junction cancer refractory to, or intolerant of, at least two previous chemotherapy regimens (ONO-4538-12, ATTRACTION-2): A randomised, double-blind, placebo-controlled, phase 3 trial. Lancet.

[6] Cunningham D (2008). Capecitabine and oxaliplatin for advanced esophagogastric cancer. N. Engl. J. Med..

[7] Van Cutsem E (2006). Phase III study of docetaxel and cisplatin plus fluorouracil compared with cisplatin and fluorouracil as first-line therapy for advanced gastric cancer: A report of the V325 Study Group. J. Clin. Oncol..

[8] Apte RS, Chen DS, Ferrara N (2019). VEGF in signaling and disease: Beyond discovery and development. Cell.

[9] Itatani Y, Kawada K, Yamamoto T, Sakai Y (2018). Resistance to anti-angiogenic therapy in cancer-alterations to anti-VEGF pathway. Int. J. Mol. Sci..

[10] Khan U, Shah MA (2019). Ramucirumab for the treatment of gastric or gastro-esophageal junction cancer. Expert Opin. Biol. Ther..

[11] Loupakis F (2011). Pharmacodynamic and pharmacogenetic angiogenesis-related markers of first-line FOLFOXIRI plus bevacizumab schedule in metastatic colorectal cancer. Br. J. Cancer.

[12] Nokihara H (2017). A phase 1 study of ramucirumab in Japanese patients with advanced solid tumors. Jpn. J. Clin. Oncol..

[13] Bocci G, Man S, Green SK, Francia G, Ebos JM, du Manoir JM (2004). Increased plasma vascular endothelial growth factor (VEGF) as a surrogate marker for optimal therapeutic dosing of VEGF receptor-2 monoclonal antibodies. Cancer Res..

[14] Spratlin JL (2010). Phase I pharmacologic and biologic study of ramucirumab (IMC-1121B), a fully human immunoglobulin G1 monoclonal antibody targeting the vascular endothelial growth factor receptor-2. J. Clin. Oncol..

[15] Takahari D (2018). Plasma biomarker analysis of ramucirumab in Japanese patients with advanced gastric cancer. J. Clin. Oncol..

[16] Van Cutsem E (2012). Bevacizumab in combination with chemotherapy as first-line therapy in advanced gastric cancer: A biomarker evaluation from the AVAGAST randomized phase III trial. J. Clin. Oncol..

[17] Mashima T (2005). p53-defective tumors with a functional apoptosome-mediated pathway: A new therapeutic target. J. Natl. Cancer Inst..

[18] Kumar V (2010). Global lymphoid tissue remodeling during a viral infection is orchestrated by a B cell-lymphotoxin-dependent pathway. Blood.

[19] Lu R, Kujawski M, Pan H, Shively JE (2012). Tumor angiogenesis mediated by myeloid cells is negatively regulated by CEACAM1. Cancer Res..

[20] Suenaga M (2016). Serum VEGF-A and CCL5 levels as candidate biomarkers for efficacy and toxicity of regorafenib in patients with metastatic colorectal cancer. Oncotarget.

[21] Loupakis F (2007). Vascular endothelial growth factorlevels in immunodepleted plasma of cancer patients as a possible pharmacodynamic marker for bevacizumab activity. J. Clin. Oncol..

[22] Azzariti A (2016). Total and not bevacizumab-bound vascular endothelial growth factor as potential predictive factors to bevacizumab-based chemotherapy in colorectal cancer. World. J. Gastroenterol..

[23] Mashima T (2019). In silico chemical screening identifies epidermal growth factor receptor as a therapeutic target of drug-tolerant CD44v9-positive gastric cancer cells. Br. J. Cancer.

[24] Hirashima K, Migita T, Sato S, Muramatsu Y, Ishikawa Y, Seimiya H (2013). Telomere length influences cancer cell differentiation in vivo. Mol. Cell. Biol..

[25] Ferrara N (2004). Vascular endothelial growth factor: Basic science and clinical progress. Endocr. Rev..

[26] Batlle E, Clevers H (2017). Cancer stem cells revisited. Nat. Med..

[27] Jiang H (2020). Jagged1-Notch1-deployed tumor perivascular niche promotes breast cancer stem cell phenotype through Zeb1. Nat. Commun..

[28] Zhao L (2017). MeCP2 promotes gastric cancer progression through regulating FOXF1/Wnt5a/beta-Catenin and MYOD1/Caspase-3 signaling pathways. EBioMedicine.

[29] Van Cutsem E, Paccard C, Chiron M, Tabernero J (2020). Impact of prior bevacizumab treatment on VEGF-A and PlGF levels and outcome following second-line Aflibercept treatment: Biomarker post hoc analysis of the VELOUR Trial. Clin. Cancer Res..

[30] Lieu CH (2013). The association of alternate VEGF ligands with resistance to anti-VEGF therapy in metastatic colorectal cancer. PLoS ONE.

[31] Hirashima Y (2009). Impact of vascular endothelial growth factor receptor 1, 2, and 3 expression on the outcome of patients with gastric cancer. Cancer Sci..

[32] Yao J (2011). Expression of a functional VEGFR-1 in tumor cells is a major determinant of anti-PlGF antibodies efficacy. Proc. Natl. Acad. Sci. U S A.

[33] Barleon B, Sozzani S, Zhou D, Weich HA, Mantovani A, Marmé D (1996). Migration of human monocytes in response to vascular endothelial growth factor (VEGF) is mediated via the VEGF receptor flt-1. Blood.

[34] Lee YJ (2010). Differential effects of VEGFR-1 and VEGFR-2 inhibition on tumor metastases based on host organ environment. Cancer Res..

[35] Freire Valls A (2019). VEGFR1(+) metastasis-associated macrophages contribute to metastatic angiogenesis and influence colorectal cancer patient outcome. Clin. Cancer Res..

[36] Hiratsuka S (2002). MMP9 induction by vascular endothelial growth factor receptor-1 is involved in lung-specific metastasis. Cancer Cell.

[37] Hicklin DJ, Ellis LM (2005). Role of the vascular endothelial growth factor pathway in tumor growth and angiogenesis. J. Clin. Oncol..

[38] Horikawa N (2017). Expression of vascular endothelial growth factor in ovarian cancer inhibits tumor immunity through the accumulation of myeloid-derived suppressor cells. Clin. Cancer Res..

[39] Hansen W (2012). Neuropilin 1 deficiency on CD4+Foxp3+ regulatory T cells impairs mouse melanoma growth. J. Exp. Med..

[40] Liu J (2020). Immune suppressed tumor microenvironment by exosomes derived from gastric cancer cells via modulating immune functions. Sci. Rep..

[41] Michalak EM, Burr ML, Bannister AJ, Dawson MA (2019). The roles of DNA, RNA and histone methylation in ageing and cancer. Nat. Rev. Mol. Cell. Biol..

[42] Azad NS (2008). Combination targeted therapy with sorafenib and bevacizumab results in enhanced toxicity and antitumor activity. J. Clin. Oncol..

[43] Sharma S (2010). A phase I study of axitinib (AG-013736) in combination with bevacizumab plus chemotherapy or chemotherapy alone in patients with metastatic colorectal cancer and other solid tumors. Ann. Oncol..

[44] Mittal K (2014). Dual VEGF/VEGFR inhibition in advanced solid malignancies: Clinical effects and pharmacodynamic biomarkers. Cancer Biol. Ther..

[45] Kummar S (2011). Phase I trial of vandetanib and bevacizumab evaluating the VEGF and EGF signal transduction pathways in adults with solid tumours and lymphomas. Eur. J. Cancer.

[46] Liu S, Nikanjam M, Kurzrock R (2016). Dosing de novo combinations of two targeted drugs: Towards a customized precision medicine approach to advanced cancers. Oncotarget.

[47] Fukahori M, Shibata M, Hamauchi S, Kasamatsu E, Machii K (2021). A retrospective cohort study to investigate the incidence of cancer-related weight loss during chemotherapy in gastric cancer patients. Support Care Cancer.

[48] Terawaki K (2017). Development of ghrelin resistance in a cancer cachexia rat model using human gastric cancer-derived 85As2 cells and the palliative effects of the Kampo medicine rikkunshito on the model. PLoS ONE.

[49] Klose R (2016). Targeting VEGF-A in myeloid cells enhances natural killer cell responses to chemotherapy and ameliorates cachexia. Nat. Commun..

[50] Osada T, Patel SP, Hammond SA, Osada K, Morse MA, Lyerly HK (2015). CEA/CD3-bispecific T cell-engaging (BiTE) antibody-mediated T lymphocyte cytotoxicity maximized by inhibition of both PD1 and PD-L1. Cancer Immunol. Immunother..

[51] Sartore-Bianchi A (2016). Dual-targeted therapy with trastuzumab and lapatinib in treatment-refractory, KRAS codon 12/13 wild-type, HER2-positive metastatic colorectal cancer (HERACLES): A proof-of-concept, multicentre, open-label, phase 2 trial. Lancet Oncol..

