# Supplementary material for: Neutralization of the induced VEGF-A potentiates the therapeutic effect of an anti-VEGFR2 antibody on gastric cancer in vivo
Source: Sci Rep. 2021 Jul 23;11:15125. doi: 10.1038/s41598-021-94584-9 (PMC8302577; doi:10.1038/s41598-021-94584-9)
Supplement: Supplementary file 1 — Supplementary Information. [file 41598_2021_94584_MOESM1_ESM.pdf]

Neutralization of the induced VEGF-A potentiates the therapeutic effect of an anti-VEGFR2 antibody on gastric cancer *in vivo*

Tetsuo Mashima, Takeru Wakatsuki, Naomi Kawata, Myung-Kyu Jang, Akiko Nagamori, Haruka Yoshida, Kenichi Nakamura, Toshiro Migita, Hiroyuki Seimiya, and Kensei Yamaguchi

Supplementary Materials and Methods

Experimental conditions and procedures of mouse xenograft study

Study design

Experimental groups: Upon subcutaneous injection of human gastric cancer cells, mice were divided into 4 groups (five - eight mice per group) and treated with vehicle, anti-VEGFR2 antibody, anti-VEGF-A antibody, or anti-VEGFR2 antibody + anti-VEGF-A antibody. The cohorts came from 2 repeated experiments.

Experimental unit: 6-week-old BALB/c-nu/nu mice (female).

Experimental procedures

Injection of human gastric cancer cells: MKN45 cells (3×10^6 cells/mouse) were suspended in 50 μL Hanks' balanced salt solution (HBSS) and were implanted subcutaneously in the right flanks of 6-week-old BALB/c-nu/nu mice.

Treatment: Therapeutic experiments (five - eight mice per group) were started approximately 14 days after implantation when tumors reached 100–200 mm^3, as measured with calipers (day 0). The anti-mouse VEGFR2 antibody (DC101) (10 or 20 mg/kg), anti-mouse VEGF-A antibody (2G11-2A05) (5 mg/kg), and vehicle (PBS) were administered intraperitoneally twice a week for 2 weeks.

Weighting: Body weight and tumor size were measured during and after treatment every week. The length (L) and width (W) of the tumor mass was measured, and the tumor volume (TV) was calculated as: $TV = (L\times W^2)/2$.

Euthanasia: At the end of the experiments, mice were euthanized by cervical dislocation.

Experimental animals

6-week-old BALB/c-nu/nu mice (female) (Charles River Laboratories, Japan), weight 15-25g.

Housing and husbandry

Animal facility: Standard animal experiment room at JFCR with automatic system of temperature, humidity and light regulation (temperature: 25 + 1°C; light/dark cycle: 12/12h; humidity: 50 + 10%).

Diet: Access to food [sterilized normal diet, CE-2 (CLEA Japan, Inc., Japan)] and sterilized water.

Cage: Sterilized plastic cages.

Cage companions: 3 animals/cage.

Bedding materials: high adsorbing bedding materials without dust. Changed every week. Environmental enrichment was done with sterile materials.

Sample size

5-8 mice/group (26 mice totally). We determined the sample size based on our previously-performed successful *in vivo* studies[23].

Allocating animals to experimental groups

Mice were divided into the above-mentioned 4 groups after randomization.

Experimental outcomes

1. To determine whether anti-VEGFR2 antibody + anti-VEGF-A antibody treatment could enhance the antitumor efficacy of anti-VEGFR2 antibody or anti-VEGF-A antibody *in vivo*.

2. To examine whether anti-VEGFR2 antibody + anti-VEGF-A antibody treatment would exacerbate the toxicity of anti-VEGFR2 antibody or anti-VEGF-A antibody.

3. To evaluate intra-tumor molecular changes after the anti-VEGFR2 antibody + anti-VEGF-A antibody treatment.

Statistical methods

Statistical analysis was performed using ANOVA, followed by the Tukey–Kramer post-hoc test.

Immunohistochemistry

Xenograft tumor samples were obtained on day 14 after the start of each treatment and formalin-fixed, paraffin-embedded tissues were prepared as described in Materials and Methods. After deparaffinization and heat-induced epitope retrieval, the sections were

incubated with rabbit anti-human Ki67 antibody (Abcam, Cambridge, UK) at 4°C overnight. The Liquid DAB+Substrate Chromogen System K3468 (Agilent Technologies (Dako)) was used for detection.

Supplementary Reference

1. Kanehisa, M., Goto, S., Kawashima, S. & Nakaya, A. The KEGG databases at GenomeNet. *Nucleic Acids Res*. **30**, 42-46 (2002).

Supplementary Figure Legends

Supplementary Fig. 1

Detection of human VEGF-A (hVEGF-A) or mouse VEGF-A (mVEGF-A) levels by Enzyme-Linked Immunosorbent assays (ELISAs) using species-specific antibodies. Human (A) and mouse (B) VEGF-A levels were measured as described in Materials and Methods with serially diluted purified human and mouse VEGF-A protein solutions (15.6–250 pg/ml).

The figures were generated by Microsoft Powerpoint (16.16.27) (https://www.microsoft.com/ja-jp/microsoft-365/powerpoint).

Supplementary Fig. 2

(A) Time course analysis of plasma murine VEGF-A induction after anti-VEGFR2 antibody treatment *in vivo*. The anti-VEGFR2 antibody (10 mg/kg) was administered intraperitoneally at day 0. At 0, 24, 48, and 72 h after treatment, mouse plasma was collected (N=3), and the murine VEGF-A concentration was measured as described in Materials and Methods. (B)–(D) Alterations in murine placental growth factor (PlGF), VEGF-C, and VEGF-D levels in mouse plasma following anti-VEGFR2 antibody administration were shown. BALB/c nude mice were injected with MKN45 cells, and mice were treated intraperitoneally with the vehicle (PBS) or anti-VEGFR2 antibody (20 mg/kg) as described in Fig. 2. At 14 days after the start of treatment, mouse plasma was collected, and murine PlGF, VEGF-C, and VEGF-D concentrations were measured as described in Materials and Methods.

The figures were generated by Microsoft Powerpoint (16.16.27) (https://www.microsoft.com/ja-jp/microsoft-365/powerpoint).

Supplementary Fig. 3

Immunohistochemcal staining of Ki67 in MKN45 xenograft tumor tissues after each treatment. Xenograft tumor samples were obtained on day 14 after the start of each treatment. Typical staining results were shown. % of Ki67-positive cells were counted in triplicate samples and calculated as in Supplementary Table 1.

The figures were generated by Microsoft Powerpoint (16.16.27) (https://www.microsoft.com/ja-jp/microsoft-365/powerpoint).

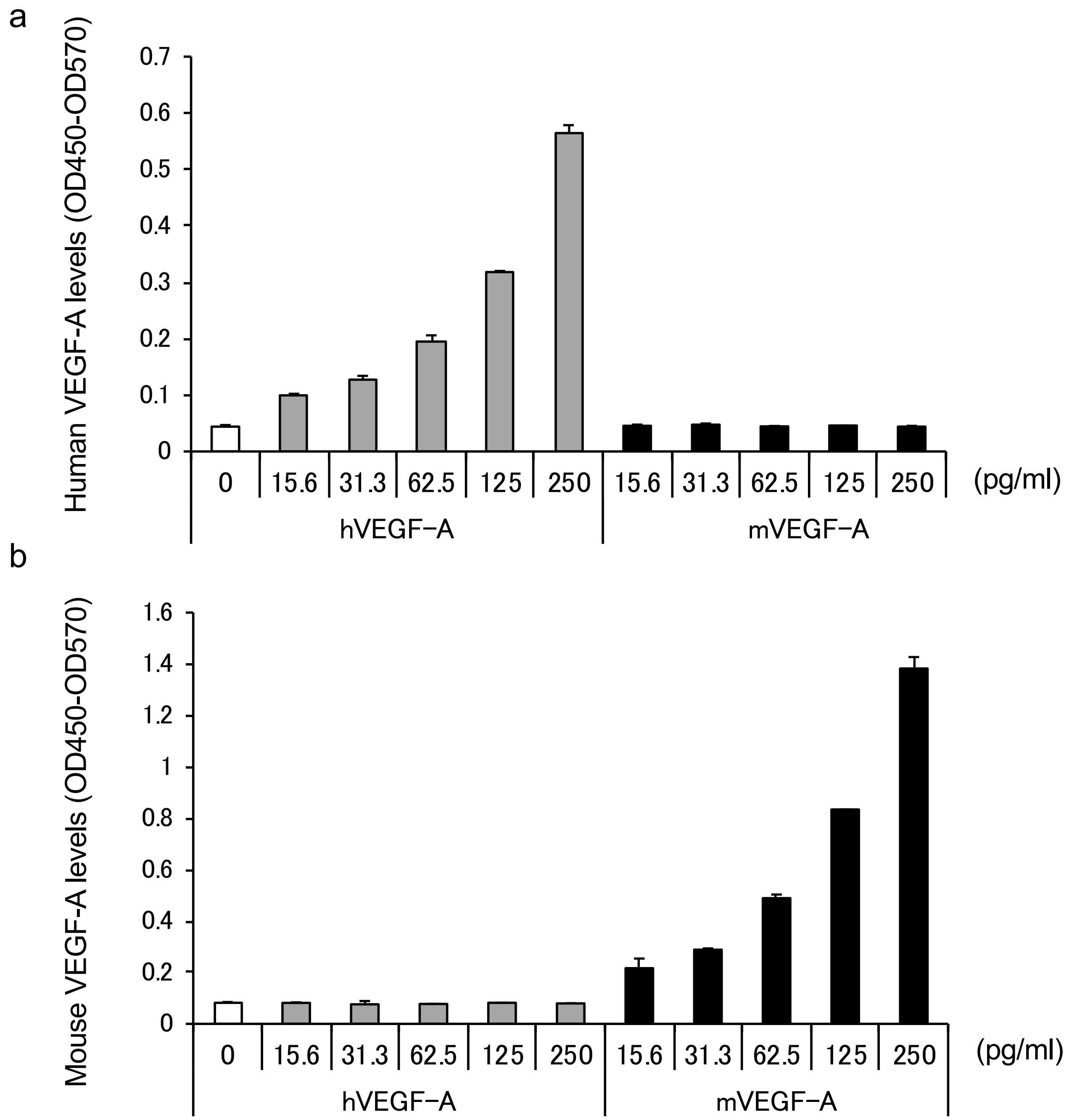

Mashima *et al.* Suppl Fig. 1

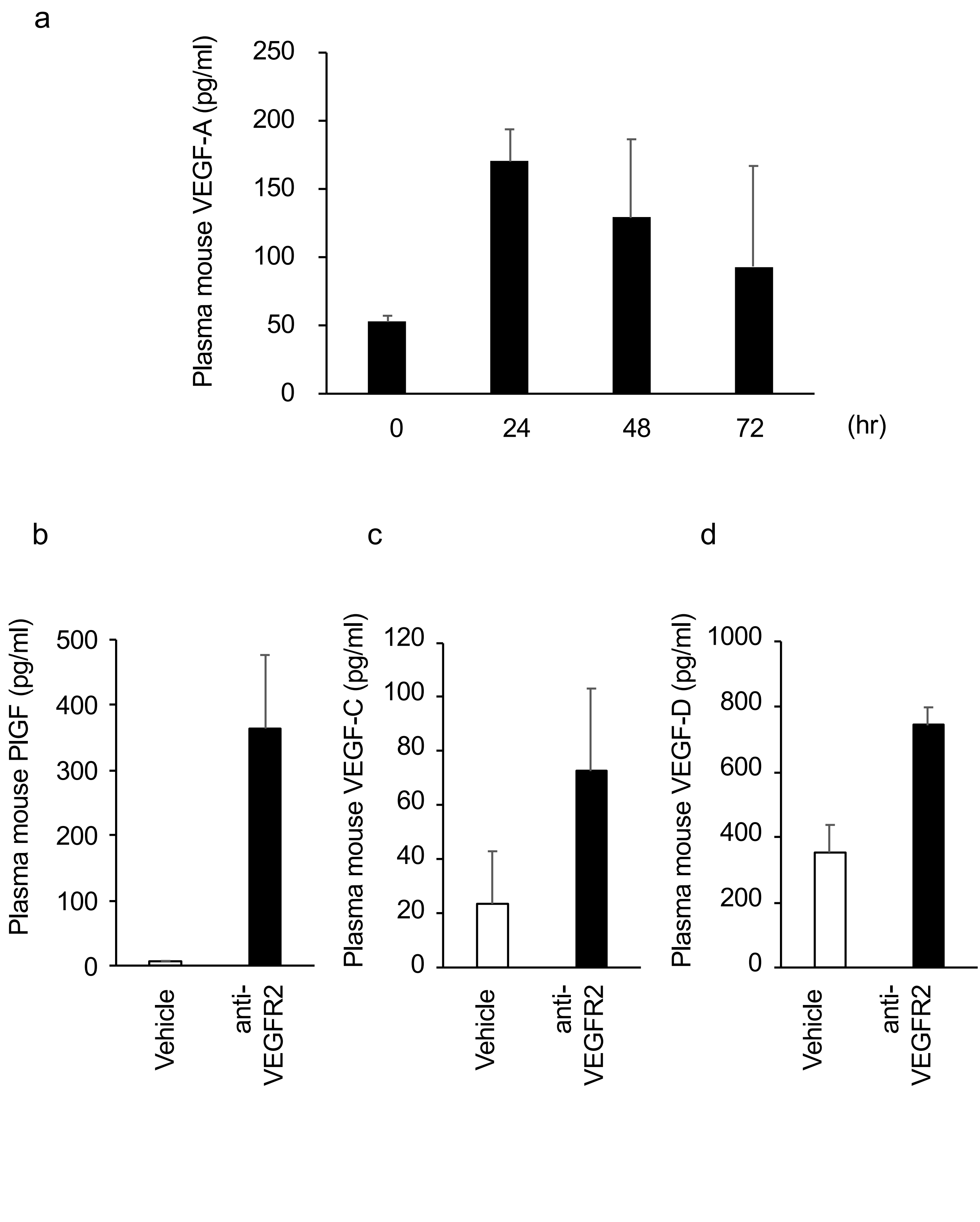

Mashima *et al.* Suppl Fig. 2

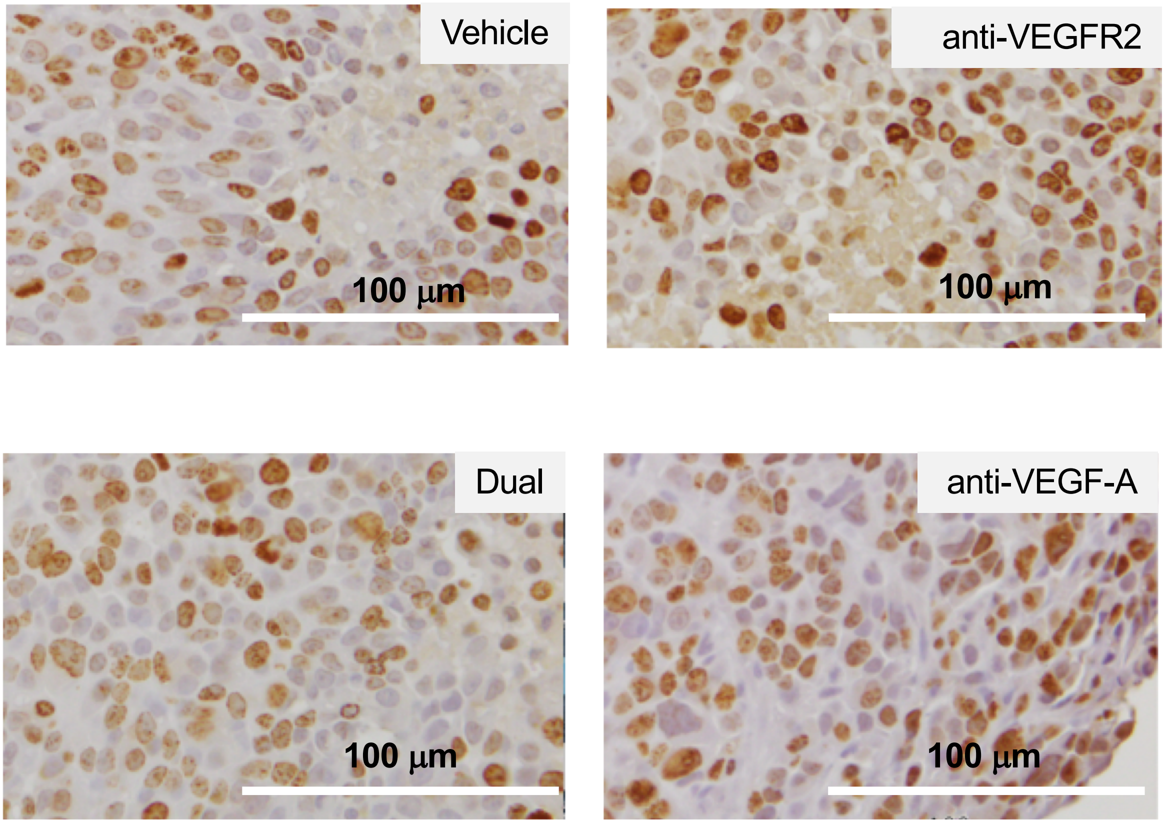

Mashima *et al.* Suppl Fig. 3

Suppl Table1 Ratio of Ki67-positive cancer cells in MKN45 xenograft tumors after VEGFR2 and VEGF-A targeting therapy

Treatments	% Ki-67(+)
Vehicle	73.9 + 5.8
anti-VEGFR2	69.8 + 9.8
Dual	50.0+ 15.8
anti-VEGF-A	53.9 + 7.2

Immunohistochemcal staining of Ki67 in MKN45 xenograft tumor tissues after each treatment was performed as described in Supplementary Fig.3. The numbers of Ki67-positive cells were counted in triplicate samples.

Suppl Table2 Pathological analysis of mouse tissues after treatment with anti-VEGFR2 and anti-VEGF-A antibodies

		Vihicle				anti-VEGFR2					Dual				anti-VEGF-A		
		#1	#2	#3	#4	#1	#2	#3	#4	#5	#1	#2	#3	#4	#1	#2	#3
Kidney	Infarction	-	±	-	-	+	-	-	±	+	+	-	-	-	-	-	2+
	Atrophic tubules	-	-	-	-	-	-	-	-	-	-	±	-	-	-	-	-
Liver	Bile duct hyperplasia	-	-	-	-	-	-	-	-	-	-	-	-	-	-	-	±

Pathological analysis in the treated mouse kidney was done on periodic acid-Schiff (PAS) stained samples and the analysis in the liver was done with hematoxylin and eosin (H&E) staining samples. (-), not observed, (±), marginal, (+), weak, (2+), medium, (3+), strong

Suppl Table3 Enriched annotation clusters (Gene Ontology) related to the genes upregulated by anti-VEGFR2 antibody treatment

Annotation Cluster 1 Enrichment Score: 3.57

Term		PValue
GO:0048255	mRNA stabilization	0.0000010
GO:0003730	mRNA 3'-UTR binding	0.0038201
GO:0045727	positive regulation of translation	0.0050826

Annotation Cluster 2 Enrichment Score: 1.83

Term		PValue
GO:0004674	protein serine/threonine kinase activity	0.0040873
GO:0005524	ATP binding	0.0208823
GO:0006468	protein phosphorylation	0.0383111

DAVID analysis (https://david.ncifcrf.gov/summary.jsp) on the genes upregulated by anti-VEGFR2 antibody treatment (classified as #2 in Fig. 5(A)) was performed. For the analysis, we extracted and analyzed the gene sets that were >60% upregulated by anti-VEGFR2 antibody treatment but not by the anti-VEGF-A antibody compared with the vehicle control. Functional annotation clustering of Gene Ontology (GO) terms was performed and enriched clusters (enriched score>1.5) were shown.

Suppl Table4 Enriched annotation clusters (KEGG pathway) related to the genes upregulated by anti-VEGFR2 antibody treatment

Annotation Cluster 1 — Enrichment Score: 1.06

Term	PValue
hsa04068:FoxO signaling pathway	0.0067908
hsa04550:Signaling pathways regulating pluripotency of stem cells	0.2604450
hsa05142:Chagas disease (American trypanosomiasis)	0.3801145

Annotation Cluster 2 — Enrichment Score: 1.04

Term	PValue
hsa05223:Non-small cell lung cancer	0.0053154
hsa04068:FoxO signaling pathway	0.0067908
hsa04910:Insulin signaling pathway	0.0301066
hsa05205:Proteoglycans in cancer	0.0404687
hsa04510:Focal adhesion	0.0456465
hsa05214:Glioma	0.0487006
hsa05212:Pancreatic cancer	0.0487006
hsa04917:Prolactin signaling pathway	0.0604197
hsa05220:Chronic myeloid leukemia	0.0624907
hsa04722:Neurotrophin signaling pathway	0.0650015
hsa04012:ErbB signaling pathway	0.0973130
hsa05215:Prostate cancer	0.0998686
hsa04915:Estrogen signaling pathway	0.1296779
hsa05213:Endometrial cancer	0.1400123
hsa04664:Fc epsilon RI signaling pathway	0.2123093
hsa04010:MAPK signaling pathway	0.2156589
hsa05200:Pathways in cancer	0.2279583
hsa05160:Hepatitis C	0.2366476
hsa05206:MicroRNAs in cancer	0.2946965
hsa04014:Ras signaling pathway	0.3202390
hsa04660:T cell receptor signaling pathway	0.3618880
hsa04062:Chemokine signaling pathway	0.6870961

DAVID analysis (https://david.ncifcrf.gov/summary.jsp) on the genes upregulated by anti-VEGFR2 antibody treatment (classified as #2 in Fig. 5(A)) was performed. For the analysis, we extracted and analyzed the gene sets that were >60% upregulated by anti-VEGFR2 antibody treatment but not by the anti-VEGF-A antibody compared with the vehicle control. Functional annotation clustering of KEGG pathways (Suppl. Ref.1) was performed and enriched clusters (enriched score >1.0) were shown.
